# Genetically Predicted 12,13-DiHOME Mediates the Association between TNF-Related Apoptosis-Inducing Ligand and Immune Thrombocytopenia

**DOI:** 10.2174/0118715303462239260413042004

**Published:** 2026-04-30

**Authors:** Chendong Jiang, Yiwen Chen, Jun Liang, Yang Feng, Zhanyan Gao, Zhan Sun, Jie Wang

**Affiliations:** 1 Department of Laboratory Medicine, Third Affiliated Hospital of Zhengzhou University, Zhengzhou, China;; 2 Department of Dermatology, Huashan Hospital, Fudan University, China

**Keywords:** Mendelian randomization, immune thrombocytopenia, 12,13-DiHOME, TNF-related apoptosis-inducing ligand, bayesian weighted Mendelian randomization, plasma metabolites

## Abstract

**Introduction:**

The role of Tumor Necrosis Factor (TNF)-Related Apoptosis-Inducing Ligand (TRAIL) has been highlighted in autoimmune and metabolic disorders. However, there is still an inadequate explanation of its causal relationship with primary immune thrombocytopenia (ITP). This study employed Mendelian Randomization (MR) to identify the potential causal associations among TRAIL, plasma metabolites, and ITP.

**Methods:**

This study was conducted focusing on analyzing data from a Genome-Wide Association Study (GWAS) comprising inflammatory proteins, plasma metabolites, and the FinnGen consortium. The Inverse Variance Weighted (IVW) method was adopted to derive the causal estimates primarily. Sensitivity analyses included Cochran’s Q test for heterogeneity, MR-Egger regression for horizontal pleiotropy, leave-one-out analysis, and validation *via* Bayesian Weighted MR (BWMR). In addition, the identification and quantification of potential mediating metabolites were realized through a two-step MR approach.

**Results:**

Genetically predicted higher TRAIL levels were associated with a reduced risk of ITP (IVW odds ratio [OR] = 0.78, 95% confidence interval [CI]: 0.65–0.92, *p* = 0.0042), which was confirmed by BWMR (OR = 0.77, 95% CI: 0.65–0.92, *p* = 0.0043). Reverse MR analysis revealed no significant causal effect of ITP on TRAIL. Among the 48 plasma metabolites causally associated with TRAIL, only 12,13-dihydroxyoctadecenoic acid (12,13-DiHOME) exhibited a significant association with ITP in BWMR (OR = 0.75, 95% CI: 0.58–0.97, *p* = 0.0316). Mediation analysis suggested that 12,13-DiHOME might partially mediate the effect of TRAIL on ITP, with an exploratory estimated mediation proportion of 7.55%.

**Discussion:**

Mediation analysis revealed that 12,13-DiHOME mediated 7.55% of the TRAIL-related ITP risk reduction. These findings provide preliminary evidence supporting a potential mediating role of 12,13-DiHOME in the protective effect of TRAIL against ITP, suggesting the existence of an immunometabolic pathway linking TRAIL to ITP pathogenesis.

**Conclusion:**

This MR study provides genetic evidence of the potentially protective causal effect of TRAIL against ITP, which may be mediated partially by 12,13-DiHOME. Our findings may highlight a novel immunometabolic pathway in ITP pathogenesis, meriting further investigation.

## 1 INTRODUCTION

Immune thrombocytopenia (ITP) is a prevalent immune-mediated hemorrhagic disorder characterized by isolated thrombocytopenia and bleeding symptoms, with an incidence of 2-10 per 100,000 individuals globally [1-3]. Its clinical burden is further amplified by the resultant complications such as intracranial hemorrhage, pregnancy-associated ITP worsening, neonatal ITP, and treatment-agnostic arterial thromboembolic events [4-9]. The pathogenesis of ITP involves complex interactions between humoral and cellular immunity, including autoantibody-mediated platelet destruction, impaired megakaryocyte maturation, and hepatic clearance induced by platelet desialylation [10-13]. Although conventional therapies are effective in more than two-thirds of patients, a subset of individuals either respond poorly to these treatments or fail to achieve long-term remission [14]. This has prompted multi-dimensional investigations into the pathogenesis of ITP, with the goal of developing novel therapies targeting relevant pathways—such as the crosstalk between inflammatory mediators and metabolic pathways. Tumor Necrosis Factor (TNF)-Related Apoptosis-Inducing Ligand (TRAIL), a key member of the TNF superfamily, has emerged as a critical regulator in autoimmune diseases. Given its dual roles, TRAIL, on one hand, can selectively induce apoptosis of pathologically activated immune cells (*e.g*., autoreactive T cells) to maintain immune homeostasis, which has been validated in systemic lupus erythematosus and multiple sclerosis [15-18]; on the other hand, dysregulated TRAIL expression may trigger autoimmune pathogenesis—for instance, reduced soluble TRAIL levels in patients with type 1 diabetes correlate with enhanced islet β-cell destruction [19, 20]. Despite these insights, research on TRAIL in ITP remains scarce. In particular, it is largely limited by methodological constraints. Specifically, existing studies are primarily observational or preclinical (*e.g*., *in vitro* megakaryocyte cultures and RAIL-deficient mouse models), which are unable to establish causal relationships due to confounding factors (*e.g*., concurrent inflammation, medication use) [21, 22]. Moreover, while TRAIL enables the modulation of lipid metabolism and inflammatory metabolite production in other diseases (*e.g*., sepsis [23]), we know little about whether it influences ITP risk through the regulation of specific plasma metabolites (*i.e.*, “inflammatory mediator-metabolite-ITP” axis).

Dysregulation of the immunometabolic network has been recognized as a key pathogenic mechanism in autoimmune diseases, in which the bidirectional regulation between inflammatory factors and metabolites is pivotal for disease progression [24]. Inflammatory factors can modulate immune cell functions by regulating their metabolic phenotypes. For instance, IL-2 and IL-1β can promote glycolysis in effector T cells (*e.g*., Th17 cells) *via* the mTOR-HIF1α pathway, while Transforming Growth Factor-Beta (TGF-β) can inhibit glycolytic activity by inducing metabolically quiescent Treg cells [25-27]. Moreover, metabolites can exert cytokine-like effects to regulate inflammation; for example, succinate and lactate can mediate inflammatory signaling, while glutathione and polyamine pathways are associated with pro-inflammatory cytokine levels [24, 28]. This regulatory axis has been validated in multiple sclerosis, Behcet's syndrome, asthma, *etc*. [29-31]. Concerning ITP, gut microbiota-derived metabolites can mediate its pathogenesis by disrupting intestinal barrier integrity and modulating immune cell differentiation and cytokine secretion [32]. Additionally, ITP patients were examined with cytokine imbalance and abnormal Trimethylamine N-oxide levels [33]. However, there is still a lack of systematic research on the direct causal associations and regulatory pathways between specific inflammatory factors (*e.g*., TRAIL) and metabolites in ITP. Notably, TRAIL regulates lipid metabolism and the release of inflammatory metabolites in other diseases [23]. Nevertheless, it remains to be urgently clarified whether it exerts its effects in ITP by remodeling the plasma metabolite profile.

Recent advances in Mendelian Randomization (MR) and Genome-Wide Association Study (GWAS) have enabled robust causal inference by leveraging genetic variants as Instrumental Variables (IVs), avoiding reverse causation and residual confounding [34, 35]. Meanwhile, the availability of large-scale GWAS datasets (*e.g*., inflammatory proteins from the GWAS Catalog, ITP cases from the FinnGen Consortium) provides an unprecedented opportunity to dissect the causal role of TRAIL in ITP and identify potential mediating metabolites. Accordingly, this study aimed to clarify the causal relationships among TRAIL, plasma metabolites, and ITP using MR.

## 2 STUDY DESIGNS AND METHODS

### 2.1 Study Design

This study employed a two-sample MR framework depending on three core assumptions as follows: (I) IVs are strongly associated with the exposure; (II) IVs are independent of any confounders affecting the exposure-outcome relationship; and (III) IVs influence the outcome only through the exposure (*i.e.*, no horizontal pleiotropy). An overview of the analytical workflow is presented in Fig. (**1**).

**Fig. (1):**
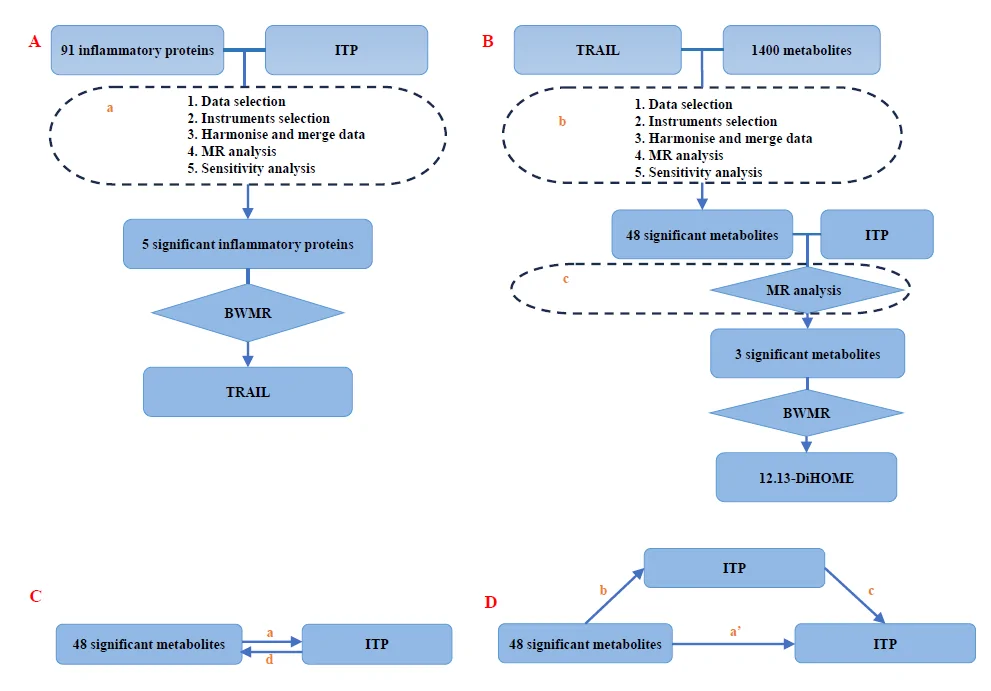
The flowchart for MR analysis. (**A**) Inflammatory proteins *vs*. ITP: 91 inflammatory proteins were regarded as exposures, and ITP as the outcome. BWMR validation identified TRAIL as the core exposure with robust correlation to ITP. (**B**) TRAIL *vs*. metabolites *vs*. ITP: TRAIL was identified to be associated with 48 metabolites; further MR/BWMR confirmed 12,13-DiHOME as the only metabolite linked to both TRAIL and ITP. (**C**) Direct TRAIL-ITP model: It illustrates the total causal effect (a) of TRAIL on ITP. (**D**) Mediation effect of 12,13-DiHOME: It quantifies the mediating role in TRAIL-ITP association. b=TRAIL→12,13-DiHOME effect; c=12,13-DiHOME→ITP effect. Indirect effect=b×c, mediation ratio=((b×c)/a) ×100%.

First, this study employed summary-level data from respective GWAS to assess the causal relationship between 91 inflammatory proteins [36] and ITP (Fig. **1A**). Furthermore, Bayesian weighted MR (BWMR) was applied to confirm initial findings, aiming at addressing potential widespread pleiotropy and polygenic architecture.

Our subsequent investigation focused on clarifying whether plasma metabolites mediated the TRAIL-ITP association (Fig. **1B**) [37]. A two-step MR approach was adopted: (1) to identify metabolites causally associated with TRAIL, and (2) and to evaluate those metabolites for a causal effect on ITP (*i.e.*, the direct causal association without considering mediation) is illustrated in Fig. (**1C**). The mediation proportion was calculated as (indirect effect / total effect) × 100%, where indirect effect = effect of TRAIL on mediator × effect of mediator on ITP, and total effect = effect of TRAIL on ITP (Fig. **1D**).

This study used de-identified data from public databases. Ethical approval was waived as the original data had undergone rigorous ethical review and approval.

Abbreviations: ITP, immune thrombocytopenia; MR, Mendelian randomization; BWMR, Bayesian weighted Mendelian randomization; TRAIL, Tumor necrosis factor-related apoptosis-inducing ligand; 12,13-DiHOME, 12,13-dihydroxy-9z-octadecenoic acid

### 2.2 Data Sources of Inflammatory Proteins, Metabolites, and ITP

Regarding the data source of metabolites, the GWAS summary statistics were cataloged in the GWAS Catalog (https://www.ebi.ac.uk/gwas/) [37], encompassing data on 1,091 metabolites and 309 metabolite ratios from 8,299 participants in the Canadian Aging Study. Of these, 850 metabolites could be defined in detail, while the remaining 241 were classified as unknown or had “partial” representation. The accession numbers for European GWASs were GCST90199621-GCST90201020.

Concerning the data for inflammatory proteins, the GWAS summary statistics for 91 proteins have been deposited in the GWAS Catalog [36]. These statistics were derived from a GWAS on protein quantitative trait loci in plasma, involving 14,824 individuals of European ancestry. The accession numbers for European GWASs were GCST90274758-GCST90274848 (https://www.ebi.ac.uk/gwas/studies/GCST90274758). For the outcomes, this study utilized data of the R10 versions from the FinnGen Consortium, comprising 405,762 controls and 882 ITP cases [38], which were made available to the public on October 31, 2022. The publicly available FinnGen database has aggregated genomic and health data for approximately 500,000 Finnish individuals (https://www.finngen.fi/en/access_results).

### 2.3 The Selection of IVs

Initially, significantly correlated Single-Nucleotide Polymorphisms (SNPs) were screened according to the threshold of *p* < 1.0 × 10^-5^ to obtain a satisfactory number of IVs. Then, the “TwoSampleMR” package was utilized to eliminate Linkage Disequilibrium (LD) by specifying the LD threshold as R^2^ < 0.001 and the aggregation distance as 10,000 kb [39]. Furthermore, during quantification using the F-statistic, F-statistic > 10 was referenced to exclude weak IVs [40]. Eventually, the Beta (B), Standard Error (SE), Allele Frequency (EAF), Sample size (N), and P-value for each SNP of exposure were extracted to calculate the F-value. The F-value was calculated as with N representing the sample size.


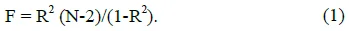


where R^2^ is calculated as


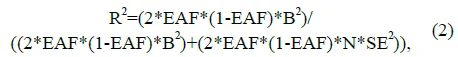


Consistent with established molecular MR practices [41-43], a relaxed SNP selection threshold (*p* < 1 × 10^−5^) was used to ensure sufficient instrument strength, given the polygenic architecture of protein and metabolite traits. Although this approach might increase the risk of weak instrument bias or false positives, several safeguards were implemented in our study: (1) an F-statistic > 10 for all IVs to minimize weak instrument bias; (2) multiple sensitivity analyses (MR-Egger, MR-PRESSO, and Cochran’s Q test) to detect horizontal pleiotropy and heterogeneity; and (3) validation *via* BWMR, a method robust to polygenic effects and widespread pleiotropy [44-46].

### 2.4 Analysis with Two-sample MR and BWMR

With genetic variants as IVs, two-sample MR was applied to infer causality under the standard three assumptions as described previously. In our analysis, the IVW method served as the primary estimator, supplemented by MR-Egger [47], weighted median [48], simple mode, and weighted mode to assess robustness [49]. BWMR represents a useful tool that incorporates a Bayesian framework to down-weight outlier variants and account for uncertainty from weak instruments. It can address challenges posed by polygenic structures and pleiotropy that may violate standard MR assumptions [44]. Therefore, BWMR was implemented in our study considering the polygenic architecture and potential widespread pleiotropy inherent to complex traits.

Furthermore, in accordance with the MR mediation analysis framework, a two-stage MR-based mediation model was employed to examine whether plasma metabolites mediate the causal pathway between TRAIL and ITP. The indirect effect of TRAIL on ITP *via* the mediator was calculated as indirect effect = b × c. The total effect of TRAIL on ITP (denoted as a) was derived from the direct MR analysis of TRAIL and ITP. The mediation proportion was computed as (indirect effect / total effect) × 100% (Fig. **1D**) [50].

### 2.5 Statistical Analysis

A significance threshold of *p* < 0.05 was used for two-sided MR tests. Sensitivity analyses included the MR-Egger intercept test for directional pleiotropy, MR-PRESSO for outlier detection and correction, and Cochran’s Q test for heterogeneity, with *p* < 0.05 indicating significant heterogeneity.

The Benjamini-Hochberg False Discovery Rate (FDR) correction to address multiple testing across the 91 inflammatory proteins, with FDR < 0.05 defined as statistically significant. However, given that the inflammatory proteins are biologically and genetically correlated, standard FDR correction may be overly conservative due to violated independence assumptions arising from shared genetic instruments (pleiotropy) and overlapping pathways, potentially obscuring true signals. Therefore, BWMR was adopted as a secondary confirmatory step for exposures revealing nominal significance in the initial IVW analysis (*p* < 0.05). In particular, BWMR is suitable for high-dimensional settings characterized by polygenic architecture and widespread pleiotropy [44]. Only exposures that were nominally significant in IVW and confirmed by BWMR (*p* < 0.05) were considered robust candidates for subsequent mediation analysis. All analyses were conducted in R (version 4.4.2) using the “TwoSampleMR” and “MR-PRESSO” packages [51]. BWMR was performed using the provided R code (Supplementary Table **1**).

## 3 RESULTS

### 3.1 Two-sample MR Analysis of Inflammatory Proteins and ITP

A two-sample MR analysis was performed to assess the causal effects of inflammatory proteins on ITP. Based on the selection criteria, 173 SNPs associated with five inflammatory proteins were included as IVs (Supplementary Table **2**). All SNPs had F-statistics > 10 (range: 19.51-1,647.73), indicating a low risk of weak instrument bias.

**Fig. (2):**
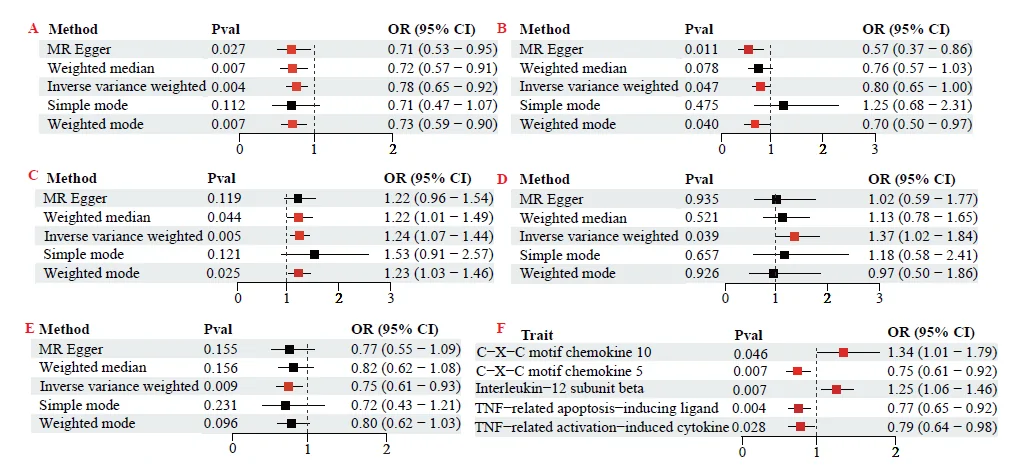
MR and BWMR Results of Inflammatory Proteins Associated with the Risk of ITP. A-E: The MR results corresponding to TRAIL (**A**), TNF-related activation-induced cytokine (**B**), Interleukin-12 subunit beta (**C**), CXC motif chemokine 10 (**D**), and CXC motif chemokine 5 (**E**) levels, respectively. (**F**) BWMR validation of the five inflammatory proteins, presenting beta values, P values, and Odds Ratios (ORs) with 95% confidence intervals (CIs). **Abbreviations:** CI, confidence interval; OR, odds ratio; and nSNP, number of single-nucleotide polymorphisms.

Initial analysis identified five inflammatory proteins nominally associated with ITP risk (*p* < 0.05) using the IVW method (Fig. **2**). Specifically, genetically predicted levels of TRAIL (Odds Ratio [OR]: 0.78; 95% CI: 0.65-0.92; *p* = 0.0042) (Fig. **2A**), TNF-related activation-induced cytokine (OR: 0.88; 95% CI: 0.79-0.97; *p* = 0.0136) (Fig. **2B**), interleukin-12 subunit beta (OR: 1.12; 95% CI: 1.02-1.24; *p* = 0.0232) (Fig. **2C**), CXC motif chemokine 10 (OR: 1.09; 95% CI: 1.01-1.19; *p* = 0.0364) (Fig. **2D**), and CXC motif chemokine 5 (OR: 0.91; 95% CI: 0.83-0.99; *p* = 0.0377) (Fig. **2E**) were associated with ITP risk. BWMR validation revealed that only TRAIL exhibited a statistically significant association with ITP (Beta: -0.2950; OR: 0.77; 95% CI: 0.65-0.92; P = 0.0043) (Fig. **2F**).

**Fig. (3):**
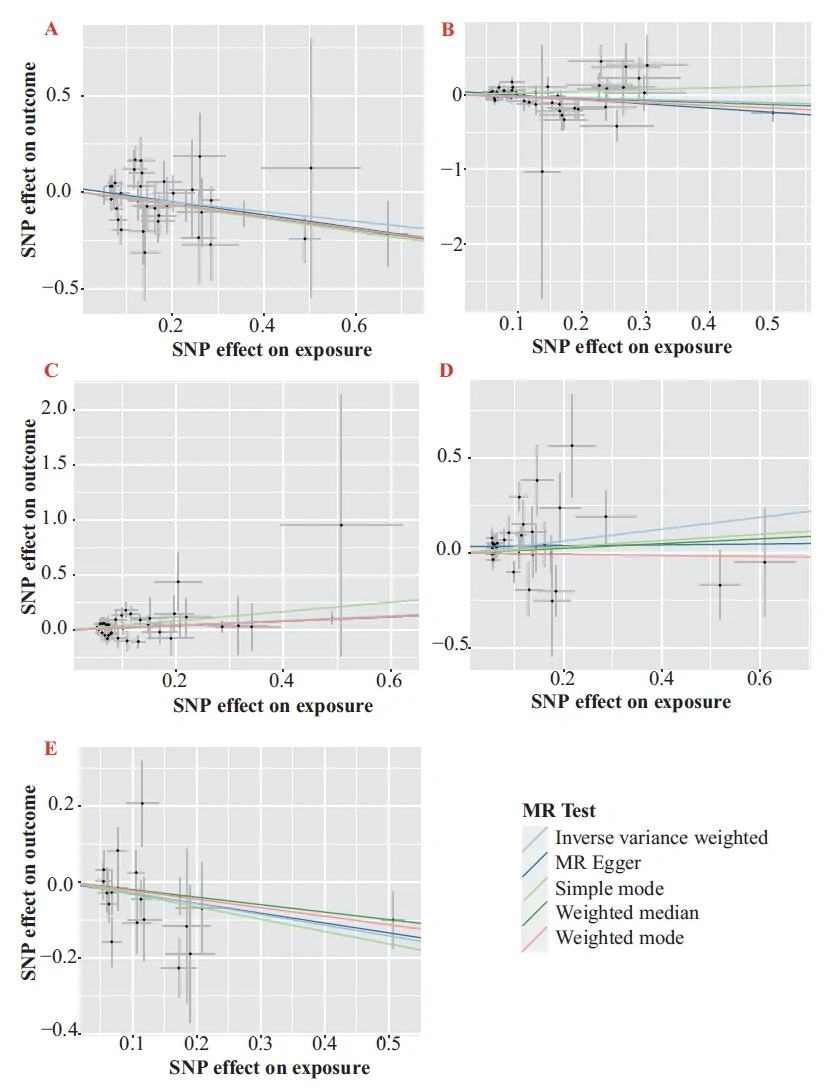
Scatter Plot of MR Analysis for Each Inflammatory Protein *vs*. ITP. Each scatter plot illustrates the causal effect of a specific inflammatory protein on ITP, with the x-axis and the y-axis representing the SNP effect on the inflammatory protein and on ITP, respectively. The exposures corresponding to each subplot are consistent with Fig. (**2**). A: TRAIL; B: TNF-related activation-induced cytokine; C: Interleukin-12 subunit beta; D: CXC motif chemokine 10; and E: CXC motif chemokine 5.

Based on MR-Egger, weighted median, simple mode, and weighted mode methods, the sensitivity analyses showed largely consistent directional effects for these proteins (Fig. **2A**- **E**). These associations were further visually supported by scatter plots (Fig. **3A**-**E**). The Cochran’s Q test indicated potential heterogeneity only for CXC motif chemokine 10 (*p* < 0.05), while MR-Egger intercept tests revealed no significant horizontal pleiotropy for any of the five proteins (*p* > 0.05). In addition, due to the potential genetic correlations among the proteins, none of these associations survived strict FDR correction (Supplementary Table **3**); thus, we proceeded to validate them using the more robust BWMR, as pre-specified in our experimental protocol. 

### 3.2 BWMR Validation of Inflammatory Proteins Associated with ITP

Subsequently, BWMR was applied to robustly validate these findings while accounting for polygenic architecture and potential pleiotropy. The associations for the other four proteins did not reach statistical significance in BWMR (all *p* > 0.05). Therefore, TRAIL was selected as the core exposure for subsequent mediation analysis. As indicated in Supplementary Table **4**, a reverse MR analysis found no significant causal effect of ITP on TRAIL levels (*P_IVW_* = 0.7885), supporting a unidirectional relationship.

### 3.3 Identification of Metabolites Mediating the TRAIL-ITP Pathway

Initially, a two-sample MR analysis of TRAIL on 1,400 plasma metabolites was implemented to explore potential mediators. It yielded the identification of 48 metabolites significantly associated with genetically predicted TRAIL levels (Supplementary Table **5**).

Next, we assessed the causal effects of these 48 metabolites on ITP. Three metabolites were nominally associated with the risk of ITP, including N2,N2-dimethylguanosine, 12,13-DiHOME, and the N-acetylputrescine to [N(1)+N(8)]-acetylspermidine ratio (Supplementary Table **6**).

Furthermore, BWMR validation (Supplementary Table **7**) confirmed that only 12,13-DiHOME showed a robust, significant association with ITP (OR: 0.75; 95% CI: 0.58-0.97; *p* = 0.0316). The remaining two metabolites did not reach statistical significance in BWMR (*p* = 0.064 and *p* = 0.0494, respectively). Consequently, 12,13-DiHOME was prioritized as a candidate mediator.

### 3.4 Causal Associations of TRAIL with 12,13-DiHOME and of 12,13-DiHOME with ITP

To assess the causal association between TRAIL and 12,13-DiHOME, MR analysis was performed using 37 SNPs as IVs. Consequently, genetically predicted higher TRAIL levels were associated with increased levels of 12,13-DiHOME (OR: 1.06; 95% CI: 1.01-1.12; *p* = 0.0295). This positive association was confirmed by BWMR (OR: 1.07; 95% CI: 1.01-1.14; *p* = 0.0163) (Supplementary Tables **8** & **9**). Moreover, it was detected with no significant heterogeneity or pleiotropy.

As for the causal association of 12,13-DiHOME with ITP, MR analysis was continued using 29 SNPs as IVs. As a result, genetically predicted higher 12,13-DiHOME levels were associated with a reduced risk of ITP (OR: 0.76; 95% CI: 0.60-0.97; *p* = 0.0290). BWMR yielded a consistent result (OR: 0.75; 95% CI: 0.58-0.97; *p* = 0.0316) (Supplementary Tables **10** & **11**), with no observation of significant heterogeneity or pleiotropy again.

### 3.5 Mediating Effect of 12,13-DiHOME in the TRAIL-ITP Association

A two-step MR mediation analysis was conducted based on the confirmed causal links: TRAIL → 12,13-DiHOME (effect *b*) and 12,13-DiHOME → ITP (effect *c*). The total effect of TRAIL on ITP (effect *a*) was -0.2526, and the indirect (mediated) effect was calculated as *b* × *c* = -0.0191, corresponding to an exploratory estimated mediation proportion of 7.55%. Therefore, 12,13-DiHOME might mediate a small portion of the protective effect of TRAIL against ITP, highlighting a potential immunometabolic pathway worthy of further investigation.

## 4 DISCUSSION

TRAIL, a pleiotropic cytokine of the TNF superfamily, is well recognized for its ability to selectively induce apoptosis in transformed or infected cells while sparing normal cells, an attribute that has been given great importance in cancer therapeutics [52-55]. Beyond this well-characterized role in oncology, TRAIL is also pivotal in immune homeostasis by selectively targeting the apoptosis of pathologically activated immune cells (*e.g*., autoreactive T cells) [15], with emerging evidence implicating its regulatory effect on autoimmune diseases [15-18]. Innovatively, our MR study provides genetic evidence supporting a causal protective role of TRAIL against ITP. Consistent with its protective effects in other autoimmune conditions (*e.g*., systemic lupus erythematosus, multiple sclerosis) [16, 17], this finding supports that reduced soluble TRAIL levels in ITP patients correlate with impaired megakaryocyte function and enhanced autoantibody production, aligning with prior observational evidence [21, 56].

Beyond its well-characterized roles in oncology and autoimmunity, TRAIL’s physiological regulation is critical for immune homeostasis, yet causal research linking it to ITP remains insufficient. Notably, a recent MR study has independently identified a causal association between TRAIL and ITP, validating this link using a similar rigorous framework [57]. Building on this, our study further uncovered a potential underlying mechanism. Critically, beyond the confirmation of the causal protective role of TRAIL against ITP, we also identified the plasma metabolite 12,13-DiHOME as a potential mediator of this effect, with an exploratory estimated mediation proportion of 7.55%. Therefore, this result suggests a novel “TRAIL-metabolite-immunity” regulatory axis relevant to the pathogenesis of ITP.

The biological plausibility of this finding has been verified by existing evidence. Soluble TRAIL levels were observed to be significantly lower in ITP patients than those of healthy controls [21, 56]. TRAIL deficiency in mouse models could induce anti-platelet antibodies and severe thrombocytopenia [15]. Besides, TRAIL exerts context-dependent regulatory effects on monocyte/macrophage activation and pro-inflammatory cytokine secretion [58, 59]: it can promote the polarization of macrophages toward a pro-inflammatory M1 phenotype to enhance anti-tumor immunity [58], while also restraining excessive activation of macrophages and reducing the secretion of pro-inflammatory cytokines (*e.g*., TNF-α, IL-1β, IL-6) to alleviate pathological autoimmunity [59]—a dual regulatory mechanism that aligns with the core pathology of ITP, where aberrant macrophage activation and dysregulated inflammatory responses contribute to autoantibody-mediated platelet destruction.

Immunometabolic crosstalk has emerged as a key regulatory axis in autoimmune diseases, with plasma metabolites—intermediates or final products of endogenous metabolic processes—serving as critical bridges linking genetic predisposition, environmental triggers, and immune dysregulation [60-63]. Concerning ITP, plasma metabolic alterations have been explored previously *via* analytical platforms such as gas chromatography-mass spectrometry [32, 61]. However, these descriptive analyses failed to disentangle causal relationships from confounding factors (*e.g*., concurrent medications, disease severity). In response to this gap and based on our prior identification of the causal protective role of TRAIL in ITP, our study specifically interrogates the causal links of TRAIL-associated plasma metabolites (derived from 1,400 evaluated metabolites) with ITP by employing MR. Notably, 12,13-DiHOME, a bioactive oxylipin, was identified as a genetically predicted protective factor against ITP. Further mediation analysis suggested that this metabolite acted as a potential mediator in the TRAIL-ITP pathway, shedding new light on the immunometabolic mechanisms underlying ITP development.

12,13-DiHOME is metabolized from linoleic acid *via* the cytochrome P450 (CYP450)-soluble epoxide hydrolase (sEH) pathway [64-67]. It has been documented to possess immunomodulatory functions, such as inhibiting monocyte pro-inflammatory cytokine production [68]. Notably, the functional role of 12,13-DiHOME is context-dependent, as it has also been reported to exert pro-inflammatory effects in certain pathological settings: elevated levels are associated with lung inflammation, ARDS, and severe COVID-19 [69], it promotes pro-inflammatory macrophage polarization and exacerbates early-life allergic inflammation [70], and it enhances NLRP3 inflammasome activation in macrophages to exacerbate inflammation [71]. This dual immunomodulatory activity is consistent with the conserved role of oxylipins in maintaining inflammatory homeostasis [72, 73]. Our MR analysis confirmed two key genetic associations: a positive correlation between TRAIL and 12,13-DiHOME levels, and an inverse correlation between 12,13-DiHOME and ITP risk. Building upon these genetic associations and existing literature, we proposed a testable hypothesis that TRAIL might upregulate the biosynthesis of 12,13-DiHOME (*e.g*., by regulating CYP450 or sEH expression [74, 75]), forming a synergistic anti-inflammatory network. In this hypothesized model, TRAIL directly inhibited immune cell overactivation, while 12,13-DiHOME further dampened pro-inflammatory responses, contributing to reduced platelet destruction in ITP collectively. Notably, this mechanistic pathway is hypothetical; our genetic study verifies the potential mediating role of 12,13-DiHOME, yet provides no direct evidence for the upstream regulatory mechanism.

## STUDY LIMITATIONS

It should be acknowledged that our MR analysis has several limitations. Firstly, the analysis was primarily based on GWAS data from populations of European ancestry, with limited representation of other ethnic groups. Therefore, it necessitates future validation in East Asian and multi-ethnic cohorts, particularly given the known ethnic differences in ITP incidence and immune-metabolic profiles. Secondly, our study failed to conduct subgroup analyses (*e.g*., by gender, age, or clinical outcome) because the summary-level data lacked the granularity needed. Thirdly, a relaxed SNP selection threshold (*p* < 1 × 10^−5^) was utilized in our study to secure a sufficient number of IVs for the proteomic and metabolomic exposures. While it is common in the field, and this study implemented multiple sensitivity analyses and BWMR validation to bolster the confidence in our findings, we cannot entirely rule out the possibility of residual bias from weak or potentially invalid instruments. Therefore, our results, particularly the initial screening of inflammatory proteins and metabolites, should be interpreted as identifying potential causal candidates that require further replication and biological validation. Finally, there was a modest estimated mediating effect of 12,13-DiHOME (~7.55%), suggesting that it explains only a portion of the protective effect of TRAIL. Consequently, findings in our study warrant replication in independent cohorts, coupled with mechanistic investigation in the future.CONCLUSION

In summary, this MR study suggests a potential causal protective role of TRAIL against ITP, which may be partially mediated by the plasma metabolite (12,13-DiHOME potentially. These findings expand the immunometabolic perspective on ITP pathogenesis and provide potential reference for screening diagnostic biomarkers and developing targeted therapeutic strategies for ITP. Future studies involving multi-population validation and functional experiments are needed to get to the bottom of the regulatory relationship and molecular mechanisms between TRAIL and 12,13-DiHOME in ITP.

## AUTHORS’ CONTRIBUTIONS

The authors confirm their contributions to the paper as follows: Y.F. and J.L.: project design and supervision, C.J. and Y.C.: Implementation; C.J., Y.C., Z.G., and Z.S.: Data collection and analysis. J.W.: Data compilation; C.J. and Y.C.: Manuscript draft; Y.F. and J.L.: Revision.

## Data Availability

The authors confirm that the data supporting the findings of this research are available within the article.

## LIST OF ABBREVIATIONS

ITP: Immune Thrombocytopenia
MR: Mendelian Randomization
BWMR: Bayesian Weighted Mendelian Randomization
TRAIL: Tnf-Related Apoptosis-Inducing Ligand
SNP: Single Nucleotide Polymorphism
CI: Confidence Interval
OR: Odds Ratio
SE: Standard Error
EAF: Effective Allele Frequency

## STANDARDS OF REPORTING

STROBE-MR guidelines and methodology were followed.
